# Drug Rash with Eosinophilia and Systemic Symptoms Syndrome Presenting After the Initiation of Staphylococcus hominis Infectious Endocarditis Treatment: A Case Report and Updated Review of Management Considerations

**DOI:** 10.7759/cureus.3679

**Published:** 2018-12-04

**Authors:** Khoa Nguyen, Mohammed S Ahmed

**Affiliations:** 1 Internal Medicine, University of Central Florida College of Medicine, Orlando, USA

**Keywords:** cutaneous adverse drug reactions, dress, endocarditis

## Abstract

We present the case of a 62-year-old Caucasian man who was being treated for mitral valve endocarditis via a six-week course of vancomycin. On Day 32 of the treatment, he developed an erythematous, pruritic, desquamating, and painful rash covering 80% of the total body surface area and intermittent fevers. Laboratory findings included leukocytosis with peripheral blood eosinophilia and elevated erythrocyte sedimentation rate, C-reactive protein, and serum creatinine.

Although the patient only completed five weeks of antibiotics, the decision was made to not complete the six-week antibiotic course due to suspicion of vancomycin-induced drug rash with eosinophilia and systemic symptoms (DRESS). The patient was then given 80 mg of intramuscular triamcinolone (Kenalog) and advised to apply topical 0.1% triamcinolone twice per day. At the three-month follow-up, the rash, leukocytosis, eosinophilia, and renal dysfunction had resolved. Clinicians must maintain a high index of suspicion for vancomycin-induced DRESS in patients with rash and eosinophilia for early recognition and treatment.

DRESS syndrome treatment typically involves discontinuing the causative drug and promptly administering steroids. However, there is a therapeutic dilemma in administering steroids during the course of an active infection. Therefore, this article serves two purposes. First, this case report highlights our approach towards managing a patient with DRESS and concurrent infectious endocarditis. Second, we include a review of the management considerations when prescribing pulsed steroids so that clinicians have a single source as a practical guide towards reducing the potentially severe systemic sequelae in DRESS syndrome and its associated treatment.

## Introduction

Drug rash with eosinophilia and systemic symptoms (DRESS) is a rare but potentially life-threatening condition characterized by multiorgan failure, lymphadenopathy, fever, and a diffuse rash typically two to eight weeks following the administration of a drug [1]. The most common drugs found to induce DRESS syndrome include anticonvulsants, minocycline, allopurinol, abacavir, and nevirapine [2]. Vancomycin is much less common; however, due to its abundant use in the clinical setting, it is starting to emerge as an important etiology for DRESS [2]. Registry of severe cutaneous adverse reaction (RegiSCAR) criteria appear to be the most widely used for diagnosis and includes at least three of the following seven characteristics: 1) skin eruption, 2) fever (>38.5 ^o^C), 3) lymphadenopathy in at least two sites, 4) involvement of at least one internal organ, 5) lymphocytosis (>4x10^3^/UL) or lymphocytopenia (<1.5x10^3^/UL), 6) blood eosinophilia (>10% or 700/UL), and 7) thrombocytopenia (<120x10^3^/UL) [3]. Cutaneous involvement is the most common clinical feature (70%-100% of cases) and includes a diffuse maculopapular inflammatory reaction (most common), erythroderma, and pruritic eruptions. Visceral organ involvement is the major cause of morbidity and mortality in this syndrome, with the most common organs affected in decreasing order being the: liver, kidney, lung, heart, and pancreas [4]. Immediate administration of high dose corticosteroids followed by a gradual tapering over 10 weeks is the treatment of choice. However, systemic corticosteroid use raises a therapeutic predicament during the management of infection. This case report highlights our approach for treating DRESS in a patient with infective endocarditis.

## Case presentation

Here, we present the case of a 62-year-old Caucasian man who was treated for non-ST-elevation myocardial infarction, heart failure with reduced ejection fraction, community-acquired pneumonia, and, later, Staphylococcus hominis mitral valve endocarditis. He was scheduled to receive six weeks of intravenous vancomycin, however, on Day 32 of treatment, he developed whole body pruritus. Over the next day, an erythematous macular rash appeared on the torso and spread centrifugally to the extremities, including the palms and soles. By Day 33, the patient began to have desquamation, intense pain to the skin, and intermittent fevers. The desquamating rash evolved to include 80% of the total body surface area (BSA) without bullae. Although there was no obvious mucosal involvement, the patient consistently reported ageusia. Abnormal laboratory findings included: white blood cell count (WBC) 23.6 x 10^3^/UL, peripheral blood eosinophilia 12%, erythrocyte sedimentation rate (ESR) 27 mm/hr, C-reactive protein 6.385 mg/L, and serum creatinine 2.3 mg/dL. His baseline laboratory values included WBC 10.5 x 10^3^/UL (normal, 4.5 – 11.0 x 10^3^/UL), eosinophils 2.9% (0.0 - 6.9%), ESR 11 mm/hr (0 - 22 mm/hr), and serum creatinine 1.3 mg/dL (0.6 - 1.3 mg/dL). There was no baseline C-reactive protein recorded (normal, <3.0 mg/L). Blood cultures were consistently negative. Serum alanine transaminase (ALT) and aspartate transaminase (AST) concentrations were within the normal range. The patient did not complain of any pulmonary symptoms, including cough, chest pain, or shortness of breath. The measurement of hemoglobin oxygen saturation by pulse oximetry remained above 95% throughout his hospital course.

Differential diagnosis discussion

We originally believed the diagnosis to be vancomycin-induced red man syndrome due to the presentation of an erythrodermic rash during concurrent vancomycin treatment. However, the vancomycin hypersensitivity reaction typically presents on the upper body, as opposed to the rash appearing over the 80% total BSA as seen in our patient. In addition, red man syndrome is a rate-related reaction that normally appears within minutes or hours of vancomycin infusion. Our patient had been receiving vancomycin for 32 days before manifestations of any hypersensitivity reaction and his skin continued to desquamate for two days after discontinuing vancomycin. Therefore, we did not believe the diagnosis was red man syndrome.

Due to the intermittent fevers and exfoliative rash following four weeks of drug administration, Stevens-Johnson Syndrome/Toxic Epidermal Necrolysis (SJS/TENS) was considered as well. The skin lesions in SJS/TENS typically start on the face and thorax, with sparing of the palms, soles, and scalp. Mucosal involvement in the oral, ocular, or urogenital regions presents as painful crusts, and erosions occur in 90% of SJS/TENS cases. In contrast to the epidermal manifestation of SJS/TENS, our patient’s skin lesions were the most erythrodermic on the palms and soles. In addition, our patient had no visible oral, ocular, or urogenital mucosal tissue sloughing, erythroderma, or pruritus. Anemia and lymphopenia are common in SJS/TENS, however, our patient had neither hematologic abnormalities and, rather, had leukocytosis. Due to the lack of classic dermal, mucosal, or hematologic findings in SJS/TENS, this differential diagnosis was also excluded.

The RegiSCAR Diagnosis Score (Table 1) was applied to predict the likelihood of DRESS syndrome as the leading diagnosis [3]. The patient received two points for peripheral eosinophilia of 22%. The patient’s skin rash contributed two points to the RegiSCAR score due to 80% of the total BSA rash extension and features of purpura and scaling. The lack of a skin biopsy contributed -1 point to the total RegiSCAR score. The serum creatinine levels, increasing from 1.3 mg/dL at baseline to 2.3 mg/dL, indicated renal involvement, which also contributed one point. The patient displayed many characteristic features of DRESS syndrome, leading to a RegiSCAR score of four, which correlates with a probable case of DRESS syndrome (Table 1).

**Table 1:** RegiSCAR Score Applied to Patient Registry of severe cutaneous adverse reaction (RegiSCAR)

Presentation: Symptom and Laboratory Finding	Result	Score
Fever (>38.5^o^C)	>38.5^o^C	0
Enlarged Lymph Nodes (>2 sites, >1cm)	No	0
Atypical Lymphocytes	No	0
Eosinophilia: 700 – 1499 or 10.0 – 19.9%	No	0
Eosinophilia: >1500 or >20%	22%	2
Skin Rash: Extent >50%	1	1
Skin Rash: At least 2 of: edema, infiltration, purpura, or scaling	Yes	1
Skin Rash: Biopsy suggesting DRESS	No	-1
Internal Organ Involvement: One	Yes, renal	1
Internal Organ Involvement: Two or more	No	0
Resolution in More Than 15 days	Yes	0
At Least 3 Biological Investigations Done and Negative to Exclude Alternative Diagnosis	No	0
Final score: <2 = no case; 2-3 = possible case; 4-5 = probably case; >5 = definitive case		

Treatment

Upon suspicion for drug-induced rash with eosinophilia and systemic symptoms, all antibiotics were stopped. However, for the next two days, the patient’s skin continued to desquamate, leukocytosis remained, and peripheral eosinophilia worsened, reaching a white blood cell count (WBC) of 31.5 x 10^3^/UL with 14% eosinophils. On Day 3 following antibiotic discontinuation, the creatinine had decreased to 1.6 mg/dL, but peripheral eosinophilia continued to increase to WBC 28 x 10^3^/UL with 22% eosinophils. Although the patient only completed five weeks of antibiotics, the decision was made to not complete the six-week antibiotic course. The patient was then given 80 mg of intramuscular triamcinolone (Kenalog) and topical 0.1% triamcinolone to be applied to the affected areas twice per day.

Outcome and follow-up

Two weeks after initiating steroids, there was a marked improvement in the erythema on his trunk and WBCs were down to 12.4 x 10^3^/UL with resolved eosinophilia. At this point, the patient was discharged from the hospital without antibiotics. He was instructed to continue using the topical triamcinolone and Aquaphor ointment for symptomatic treatment at home. When seen at follow-up three months later, the rash had completely resolved except mild erythema to his hands and feet. His WBC count was normal with 2.9% eosinophils (normal 0.0-6.0%). At the four-month follow-up, the patient continued to note improvement in ageusia. Given the ongoing improvement in ageusia and lack of DRESS-like symptoms, no further workup was undertaken. He was not re-challenged with vancomycin. A lymphocyte transformation test, patch test, autoantibody profiling, and cytomegalovirus (CMV) serology were not acquired due to symptom resolution in the patient. At the five-month follow-up, the patient resumed his home blood pressure medications prior to his illness, with no sequelae.

## Discussion

DRESS syndrome is a serious condition that confers a mortality rate as high as 10% [5]. Prompt diagnosis and treatment are necessary to prevent fatal outcomes. The diagnosis of DRESS syndrome should be suspected in any patient who exhibits a skin rash, lymphadenopathy, fevers, or facial edema two to six weeks following drug administration. The time latency between drug initiation and symptom onset is of particular diagnostic importance because DRESS syndrome typically occurs two to six weeks following drug intake, as compared to four to 28 days seen in SJS/TENS [1]. Laboratory evaluations are aimed at confirming the diagnosis, excluding other potential causes, and examining the extent of visceral organ involvement. A complete blood count with differential and peripheral smear can be the initial lab value indicating DRESS syndrome. Peripheral eosinophilia >700/uL or >10%, lymphocytosis, or atypical lymphocytes on peripheral smear can all suggest DRESS syndrome [3]. Visceral organ involvement is the major cause of morbidity and mortality in these patients. Serum ALT greater than twice the normal limit and/or alkaline phosphatase greater than 1.5 times the normal limit indicate hepatic damage, the most common visceral organ involved [4]. Serum creatinine greater than 1.5 times the patient’s baseline, proteinuria, or hematuria suggest severe renal damage. In patients experiencing pulmonary symptoms, such as cough or shortness of breath, a chest radiograph can show interstitial pneumonitis [4]. The RegiSCAR score and Naranjo Adverse Drug Reaction Probability Score can be calculated to determine the likelihood of DRESS syndrome and the causative agent, respectively [3,6].

Treatment of DRESS syndrome is imperative to avoid fatal outcomes. However, published guidelines for the treatment of DRESS are scarce. Treatment revolves around the immediate withdrawal of the drug and administering symptomatic treatment such as intravenous corticosteroids, antipyretics, and topical emollients [7]. Systemic corticosteroids in the form of intravenous or oral pulsed strategies are often employed. Systemic steroids should begin at a minimum dose of 1.0 mg/kg/day of prednisone or its equivalent. A gradual taper is recommended over a period of three to six months after laboratory and clinical stabilization. Significant improvement is expected within a few days of drug withdrawal and systemic corticosteroid treatment [1].

While steroids have often been the treatment of choice in DRESS, clinicians must maintain a high index of suspicion for severe complications that may arise from pulsed prednisone therapy. Immune reconstitution inflammatory syndrome (IRIS) is a collection of potentially fatal inflammatory disorders that can arise after steroid withdrawal at sites of previous infections. Cytomegalovirus (CMV) reactivation in IRIS most commonly presents as immune recovery uveitis (IRU) or immune recovery vitritis (IRV) in HIV-infected patients taking antiretroviral therapy [8]. This highlights the need for continued ophthalmologic evaluations in HIV-infected individuals who have a history of CMV retinitis, even if the patient has not required anti-CMV treatment for greater than four years. Immunocompetent hosts are susceptible to this complication as well, seeing that steroid use was found to be an independent risk factor for the development of CMV reactivation colitis in immunocompetent hosts [9]. Therefore, pulsed steroids must be used with great caution to reduce the risk of the serious complications associated with IRIS.

There are many tests that clinicians can pursue for the early identification of potentially severe systemic sequelae from pulsed prednisone therapy. Any patient presenting with a febrile illness with greater than 10% atypical lymphocytosis should raise the suspicion for mononucleosis. Clinicians should have a low threshold to test for CMV serology. Other herpesvirus serology can be included based on the clinical presentation. It is important for clinicians to monitor patients with DRESS syndrome for months to years following resolution, as autoimmune diseases, such as autoimmune thyroid disease, alopecia areata, type 1 diabetes mellitus, and autoimmune hemolytic anemia, have been reported, particularly in young patients [5]. A gradual tapering of systemic steroids over a long duration has been shown to reduce the likelihood of developing autoimmune long-term sequelae [10]. However, clinicians must balance this effect with the potential for a higher risk of opportunistic infections. Acquiring titers of autoantibodies to thyroid peroxidase, thyroglobulin, herpesviruses, and antinuclear antibody can, therefore, play a beneficial role in the prognosis of patients with DRESS.

Considering that a rechallenge with the culprit drug is contraindicated, lymphocyte transformation test (LTT) can be valuable options for identifying the causal drugs. Acquiring the LTT at the right time in the clinical course of DRESS is very important. LTT was found to be most useful in the recovery phase of DRESS, as the sensitivity and specificity of LTT in the recovery phase was found to be 73% and 82%, respectively [11]. This is in contrast to sensitivity and specificity of 40% and 30%, respectively, in the acute phase. Therefore, the optimal time for LTT is five to eight weeks after the onset of skin eruption [12]. A positive LTT within one week of skin findings could suggest other types of drug eruptions, as DRESS rarely has positive LTT results in the acute phase [12]. However, LTT is primarily indicated for identifying anticonvulsants as the causal drug in DRESS [11]. There have not been any studies to date analyzing the utility of LTT for identifying vancomycin as the culprit in DRESS, which limits its value in our patient.

Patch tests can be helpful in identifying vancomycin as the causal drug in DRESS in addition to LTT. Santiago et al. found that a positive patch test was observed in 18 out of 56 patients (32.1%), 17 of which were with antiepileptics [13]. In the antiepileptic group, 13 of the 17 medications were carbamazepine alone, outlining the use of a patch test in carbamazepine-induced DRESS syndrome. Like in LTT, few studies have identified the value in patch tests for vancomycin-induced DRESS syndrome. Patch testing has, however, been useful in DRESS for identifying drug imputability and cross-reactions [14].

There can be a therapeutic dilemma around administering steroids in the setting of active infection. In addition, there is a lack of studies establishing guidelines for the management of DRESS syndrome in the presence of active infection. One report demonstrated the successful management of DRESS syndrome in the course of infectious endocarditis involving intravenous immunoglobulins, N-acetylcysteine, montelukast, and gentamicin, while avoiding systemic corticosteroids [15]. Previous cases have been successful in discontinuing the antibiotics and starting high-dose steroid treatment following negative blood and urine cultures in osteomyelitis [16]. Randomized controlled clinical trials are lacking and are necessary for identifying optimal treatment strategies for DRESS syndrome. This case report highlights our approach in treating DRESS in a patient with infective endocarditis.

There were a few limitations to this case report. We were unable to obtain a skin biopsy due to patient refusal. Skin biopsies containing perivascular lymphocytic infiltrations, apoptotic keratinocytes, vacuolar degeneration, or epidermal necrolysis can aid in diagnosing DRESS [4]. However, our case had a RegiSCAR score of four, which correlated with a probable case of DRESS. While a skin biopsy could provide a more robust suggestion for DRESS, it is not necessary for diagnosis. This case report can still prove valuable for clinicians who do not have the resources or patient compliance to attain a skin biopsy.

In addition, there was some confusion in the cause of DRESS syndrome because the vancomycin and warfarin were started around the same time. However, vancomycin has been identified to be a common etiology for DRESS in multiple reports whereas no case reports exist for warfarin [17]. In fact, one report demonstrated warfarin tolerance in a patient with acenocoumarol-induced DRESS [18]. In our case, rash and eosinophilia resolved after stopping vancomycin and initiating steroids, and the patient continues to be on warfarin. A modified Naranjo Adverse Drug Reaction (ADR) Probability Scale can be applied to determine the likelihood of an adverse drug reaction occurring due to recent drug administration instead of as a result of other factors [6,19]. The patient exhibited a Naranjo ADR score of three, which correlates with a possible ADR due to vancomycin (Table 2). Whereas, the patient received a Naranjo ADR score of zero for warfarin, which correlates with a doubtful cause of ADR (Table 3). Therefore, it is reasonable to suggest that the ADR seen in our patient was due to vancomycin.

**Table 2:** Naranjo Adverse Drug Reaction Probability Scale for Vancomycin

Question	Answer	Score
Are there previous conclusive reports on this reaction?	Yes	1
Did the adverse event appear after the suspected drug was administered?	Yes	2
Did the adverse reaction improve when the drug was discontinued or a specific antagonist was administered?	Yes	1
Did the adverse event reappear when the drug was readministered?	Do not know	0
Are there alternative causes (other than the drug) that could on their own have caused the reaction?	Yes	-1
Did the reaction appear when a placebo was given?	Do not know	0
Was the drug detected in blood (or other fluids) in concentrations known to be toxic?	No	0
Was the reaction more severe when the dose was increased or less severe when the dose was decreased?	Do not know	0
Did the patient have a similar reaction to the same or similar drugs in any previous exposure?	Do not know	0
Was the adverse event confirmed by any objective evidence?	No	0
Scoring: 0 = doubtful Adverse Drug Reaction (ADR); 1-4 = possible ADR; 5-8 = probably ADR; >9 = definite ADR		

**Table 3:** Naranjo Adverse Drug Reaction Probability Scale for Warfarin

Question	Answer	Score
Are there previous conclusive reports on this reaction?	No	0
Did the adverse event appear after the suspected drug was administered?	Yes	2
Did the adverse reaction improve when the drug was discontinued or a specific antagonist was administered?	Do not know	0
Did the adverse event reappear when the drug was re-administered?	No	-1
Are there alternative causes (other than the drug) that could on their own have caused the reaction?	Yes	-1
Did the reaction appear when a placebo was given?	Do not know	0
Was the drug detected in blood (or other fluids) in concentrations known to be toxic?	No	0
Was the reaction more severe when the dose was increased or less severe when the dose was decreased?	Do not know	0
Did the patient have a similar reaction to the same or similar drugs in any previous exposure?	Do not know	0
Was the adverse event confirmed by any objective evidence?	No	0
Scoring: 0 = doubtful Adverse Drug Reaction (ADR); 1-4 = possible ADR; 5-8 = probably ADR; >9 = definite ADR		

Vancomycin-induced DRESS syndrome has spiked in the last five years, due to the increased utilization of vancomycin, higher vancomycin trough targets, and higher total dosages [2]. A retrospective study by Magill et al. demonstrated that parenteral vancomycin was the most commonly used antibiotic for both community-onset and health care facility-onset infections for patients in critical care and non-critical care locations [20]. The increasing use of vancomycin has raised some concerns over the facilitation of antimicrobial resistance. Highlighting the association of vancomycin with DRESS further supports its judicious use along with aiding clinicians to diagnose DRESS early in its clinical course before devastating consequences ensue. Our case report highlights that we need to maintain a high index of suspicion for DRESS in patients with rash and eosinophilia for early recognition and treatment.

## Conclusions

As vancomycin is currently the most commonly used antibiotic in community-onset and health care facility-onset infections, it is important to raise awareness in health care professionals about its potential for eliciting DRESS syndrome. Exfoliative rash two to eight weeks following the administration of a drug, fevers, lymphadenopathy, rapid deterioration of hepatic and renal function, and eosinophilia should raise one’s suspicion of DRESS. Prompt diagnosis and immediate withdrawal of the drug can reduce morbidity and mortality. In the setting of an active infection, one treatment approach can be discontinuing vancomycin and administering systemic corticosteroids pending negative blood cultures. Further studies are needed to establish specific guidelines for the management of DRESS in the presence of an active infection.

## Appendices

Patient's perspective

My whole experience with DRESS was very painful. I likened it to having my whole body dipped into scalding water. There was no part of me that was unaffected. My whole body peeled and I lost most of the hair on my skin. In the hospital, they gave me morphine, but this was not really that effective for the pain. I pretty much just had to tough it out for about a month. The worst parts were my hands and feet, which made it difficult to grasp things or walk. My hands and feet actually peeled about three separate times. The only pictures I have are ones that I took of my hands. As far as I know, nobody took pictures at the hospital, which is a little regrettable.

One of the secondary effects of DRESS was the loss of all taste senses, except bitter. To this day, I can’t taste salty or sweet. The ear, nose, and throat (ENT) doctor thinks that as well as sloughing of the outer skin, I sloughed my taste buds. Hopefully, they will grow back someday - Oh, to be able to taste a Hershey Bar or salty French fry again!

The road to recovery was long and painful. I still have dermatitis on my hands and feet. Except for not being able to taste, I am mostly recovered. I’m sure that DRESS has delayed my FULL recovery from my heart and lung problems. Although I feel pretty good now, I am only at about 70 percent of my normal strength. I miss being able to do full recreational activities such as kayaking or playing a full round of golf.
